# A systematic review of the single-stage treatment of chronic osteomyelitis

**DOI:** 10.1186/s13018-019-1388-2

**Published:** 2019-11-28

**Authors:** Bethan Pincher, Carl Fenton, Rathan Jeyapalan, Gavin Barlow, Hemant K. Sharma

**Affiliations:** Department of Trauma & Orthopaedics, Hull Royal Infirmary, Anlaby Road, Hull, HU3 2JZ UK

**Keywords:** Infection, Chronic osteomyelitis, Single stage, Surgery

## Abstract

**Background:**

Despite advances in surgery, the treatment of chronic osteomyelitis remains complex and is often associated with a significant financial burden to healthcare systems. The aim of this systematic review was to identify the different single-stage procedures that have been used to treat adult chronic osteomyelitis and to evaluate their effectiveness.

**Methods:**

Ovid Medline and Embase databases were searched for articles on the treatment of chronic osteomyelitis over the last 20 years. A total of 3511 journal abstracts were screened by 3 independent reviewers. Following exclusion of paediatric subjects, animal models, non-bacterial osteomyelitis and patients undergoing multiple surgical procedures, we identified 13 studies reported in English with a minimum follow-up period of 12 months. Data extraction and quality assessment were performed for all studies. Non-recurrence was defined as resolution of pain without recurrence of sinuses or need for a second procedure to treat infection within the described follow-up period.

**Results:**

A total of 505 patients with chronic osteomyelitis underwent attempted single-stage procedures. Following debridement, a range of techniques have been described to eliminate residual dead space including biologic and non-biologic approaches. These include musculocutaneous flaps, insertion of S53P4 glass beads or packing with antibiotic-loaded ceramic or calcium-sulphate pellets. The average follow-up ranged from 12 to 110 months. The most common organism isolated was *Staphylococcus aureus* (35.2%). Non-recurrence ranged from 0 to 100%. Debridement alone was statistically significantly inferior to approaches that included dead space management (54.5% versus 90% non-recurrence). Biologic and non-biologic approaches to dead space management were comparable (89.8% versus 94.2% non-recurrence).

**Conclusion:**

A wide range of single-stage procedures have been performed for the treatment of chronic osteomyelitis. In general, studies were small and observational with various reporting deficiencies. No one dead space management technique appears to be superior, but debridement alone that leaves residual dead space should be avoided.

## Background

Despite advances in surgery, the treatment of chronic osteomyelitis (COM) remains challenging and complex. The majority of cases are post-traumatic, with rates of infection in open long bone fractures quoted as being between 4 and 64%. Most commonly affecting young adult males, COM can lead to significant morbidity for the patient as well as negatively affecting livelihood. The management of this condition has also been shown to result in a large financial burden for the NHS due to the multiple surgeries, prolonged hospital stays and extensive courses of antibiotics that are often required for successful management [1]. The principals of surgical treatment include adequate debridement, obtaining appropriate microbiology specimens, dead space management and achieving skeletal stability with soft tissue cover. Appropriate systemic, and often local, antibiotic therapy in the post-operative period is also required to achieve eradication of infection. A multidisciplinary team approach to the treatment of these patients is therefore fundamental to achieving a successful outcome.

The concept of a single-stage procedure for the treatment of COM has been trialled in specialist centres for the last 20 years. Many different methods have been described to provide skeletal stability and obliterate dead space following debridement. Currently, there is no universal consensus or guidelines on how chronic osteomyelitis is best managed. The aim of this systematic review was to identify the different types of single-stage procedures that have been performed and reported for COM, to evaluate their effectiveness and identify factors that influence recurrence.

## Patients and methods

The study design was guided by the Preferred Reporting Items for Systematic Reviews and Meta-Analyses (PRISMA) checklist (Additional file 1). A search strategy was created to yield the maximum number of relevant results. The search was designed to capture all articles referring to osteomyelitis with a focus on treatment, management or surgery. We searched Ovid Medline and Embase databases (January 2017) for comparative and non-comparative studies from the last 20 years of single-stage procedures carried out for the treatment of COM. Duplicates were then removed.

Supplementary searches were made through Internet search engines to identify any additional articles or references to publications on chronic osteomyelitis. We also reviewed the reference list from each relevant article identified through our database search to ensure that we had captured all of the available literature within our designated time period. No other articles were identified through these channels that met our inclusion/exclusion criteria. Three thousand eleven abstracts were screened by 3 independent reviewers and excluded according to the following a priori exclusion criteria.

Exclusion criteria:

▪ Children (< 16 years)

▪ Animal models

▪ Non-bacterial osteomyelitis

▪ Non-long bone osteomyelitis

▪ Spinal involvement

▪ Treatment of infected non-unions

▪ Publications not available in English

▪ Multiple planned surgical procedures

▪ The use of external fixators for skeletal stability

▪ Less than 12 months follow-up

▪ Case series with fewer than 10 patients in total

Following this initial screening, we found 152 articles that met our inclusion criteria. The full texts of these remaining articles were reviewed to identify reports of true single-stage procedures. All patients within these studies were required to have met appropriate diagnostic criteria for chronic osteomyelitis including classical radiological features, with positive microbiology tissue cultures or histology. Articles excluded at this stage included those where multiple or staged debridements had been planned or where external fixation was utilised for stability, which would require additional procedures for adjustment or removal.

After completion of this full-text review, we had identified 13 publications that met our inclusion criteria and specifically reported on single-stage procedures for the treatment of chronic osteomyelitis. These publications were quality assessed by 2 independent reviewers using the Modified Coleman Methodology Score (MCMS) [2]. No discrepancies were found between the MCMS results calculated by the 2 reviewers. Data extraction was then performed by the lead author on patient demographics, length of follow-up, Cierny-Mader classification, microbiology and patient outcome. The primary measure of outcome for this review is non-recurrence of osteomyelitis. Non-recurrence was defined as resolution of pain with no recurrence of sinuses and no need for a second procedure to treat infection within the study’s follow-up period (at least 1-year post-surgery). The unadjusted proportions of patients without recurrence are presented by procedure type and approach to dead space management. Descriptive statistics are also presented for host status and microbiological findings. The chi-square test was used to statistically compare the proportion of patients with non-recurrence with different approaches.

## Results

The completed PRISMA flow diagram is shown below. Thirteen eligible studies were identified with 505 patients undergoing a single-stage procedure for COM within these studies. Table 1 shows the characteristics of the studies included. Patient ages ranged from 16 to 88 years (mean ages ranged from 30 to 67 years) with a ratio of 2.5 males for every 1 female. No randomised trials were identified. Eight studies were performed prospectively and five retrospectively with ten of uncontrolled observational and three of comparative observational study designs.

**Table 1:** Results by study [3–15]

Article	Total patients	M to F	Age (mean)	F/U month range (mean)	COM patients	Treatment method	Non- recurrence (%)	MCMS
Yamashita et al., Japan, 1998 [3]	18	10:8	16–77 (39)	24–75 (55)	14	Debridement + antibiotic loaded calcium-hydroxyapatite	100	72
Simpson et al., Oxford, 2001 [4]	15	39:11	16–78 (47.2)	12–48 (26)	8	Debridement: wide excision + gentamicin beads ± free flap	100	75
	29	/	16–79 (42.9)	12–48 (26)	21	Debridement: marginal excision + gentamicin beads ± free flap	71.4	/
	6	/	42–82 (67)	12–48 (26)	4	Minimal debridement	0	/
Kuokkanen et al., Helsinki, 2002 [5]	21	18:3	30–69 (34)	12–78 (36)	13	Debridement ± muscle flap	92.3	65
Hashmi et al., Sheffield, 2004 [6]	17	17:0	17–53 (37)	60–84 (70)	8	Debridement + Lautenbach	75	77
Beals et al., Oregon, 2005 [7]	30	25:5	16–80 (43.6)	24–180 (72)	7	Debridement ± muscle flap	100	60
Lu et al., China, 2006 [8]	35	28:7	16–72 (30.2)	13–55 (27.8)	26	Debridement + allograft bone grafting	92.3	63
Smith et al., Leeds, 2006 [9]	41	26:15	16–76 (45.3)	12 (12)	41	Debridement + free or local muscle flaps	95	59
Chang et al., Bologna, 2007 [10]	40	36:29	18–69 (39.8)	36–334 (75)	40	Debridement alone	60	62
	25	/	18–69 (39.8)	36–334 (75)	25	Debridement + Osteoset-T pellets	80	/
Caesar et al., Oswestry, 2009 [11]	35	26:9	19–81 (42)	24–150 (110)	23	Debridement + Lautenbach	85.3	69
Drago et al., Milan, 2013 [12]	27	18:9	20–80 (44)	12–36 (16)	24	Debridement + bioactive glass	87.5	74
Romano et al., Milan, 2014 [13]	27 (as Milan 2013)	18:9	20–80 (44)	12–36 (21.8)	24 (as Milan 2013)	Debridement + bioactive glass	87.5	71
	27	16:11	24–74 (47)	12–36 (22.1)	25	Debridement + antibiotic loaded calcium-hydroxyapatite	89.8	/
	22	14:8	23–77 (44.9)	12–36 (21.5)	20	Debridement + tricalcium-phosphate with antibiotic loaded bone matrix	88.9	/
Ferguson et al., Oxford, 2014 [14]	193	150:43	16–82 (46.1)	16–85 (44)	128	Debridement + Osteoset-T ± muscle flaps	86.4	83
McNally et al., Oxford, 2016 [15]	100	65:35	23–88 (51.6)	12–34 (19.5)	78	Debridement + CERAMENT G	97.4	79


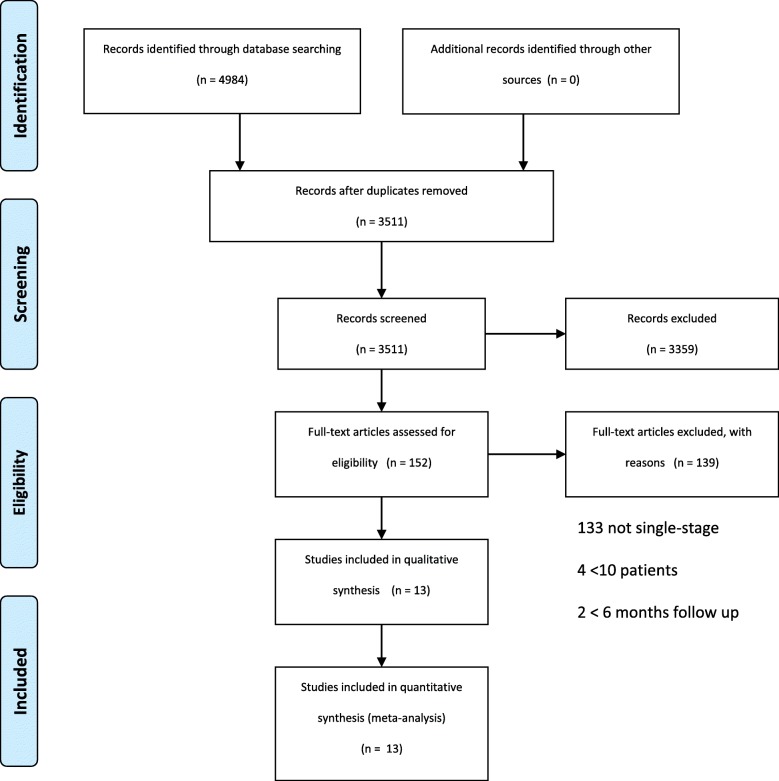


*Staphylococcus aureus* was the most common single organism isolated and was found to be the primary organism in 35.2% of patients. Just over one quarter (28.4%) of *Staphylococcus aureus* isolates were found to be methicillin-resistant (MRSA). Gram-negative organisms including *Pseudomonas spp.* accounted for 15.8% of positive microbiology samples; see Table 2. Cierny-Mader host type was A in 44% of cases, B in 54% and C in 2%. The majority of cases were classified as stage 3 or higher (79%).

**Table 2:** Microbiology results

Organism	Samples (%)
MSSA	25
MRSA	10
Gram-negative aerobes	10
CNS	7
Pseudomonas	6
Enterococci	3
Streptococci	2
Polymicrobial	19
Not identified	19

**Table 3:** Results by procedure type

Treatment method	Patients	Non-recurrence (%)
Debridement alone	44	54.50
Debridement + PMMA gentamicin beads	29	79.30
Debridement + Lautenbach	31	82.60
Debridement + Ca-Phos & Abx-loaded bone matrix	20	86.40
Debridement + bioactive glass	24	87.50
Debridement + Osteoset-T (Ca-S + tobramycin)	153	88.20
Debridement + allograft bone	26	92.30
Debridement + Abx-loaded Ca-Phos HA	39	92.90
Debridement + muscle flap	61	95.10
Debridement + cerament G (Ca-S HA + gentamicin)	78	97.40

*PMMA* polymethyl methacrylate, *Ca-S* calcium sulphate, *Ca-S HA* calcium sulphate + hydroxyapatite, *Ca-Phos HA* calcium phosphate + hydroxyapatite

The total number of patients for each individual treatment approach was small (20 to 153 patients). Reported non-recurrence rates varied from 0 to 100%. Simple debridement alone had the poorest outcome (54.5% non-recurrence versus 90%; chi-square = 45.6, *P* value < 0.05). All other approaches had a success rate of at least 79.3% (Table 3). The approach with the highest number of patients was debridement using calcium sulphate plus tobramycin (Osteoset-T) for dead space management with a non-recurrence rate of 88.2% (*N* = 153). Non-biologic approaches were more commonly reported (*N* = 343) than biologic (*N* = 87); cure rates were similar for both (89.8% versus 94.2%) respectively; chi-square = 1.63, *p* = 0.20; see Table 4.

**Table 4:** Dead space solutions and anatomical site

	Dead space management	Patients	Non-recurrence (%)
Biologic	Muscle flap	61	95.1
	Bone allograft	26	92.3
	Total	87	94.2
Non-biologic	Abx-loaded Ca-HA	117	95.7
	Abx-loaded Ca-Sulph	153	88.2
	Bioactive glass	24	87.5
	Abx-loaded Ca-phos	20	86.4
	Abx-loaded PMMA	29	79.3
	Total	343	89.8
Bone	No recurrence	Recurrence	Recurrence (%)
Tibia	290	15	4.9
Femur	195	13	6.3
Humerus	42	3	6.7
Other	23	2	8.0

Four studies did not report any data on patient host type or staging of disease. From data extracted, no patient with stage 1 or 2 disease (*N* = 167) suffered a recurrence of infection within the period of follow-up. Patients of host type C had a recurrence rate of 63.6% (*N* = 11) compared to 6.2% in host type A and 9.4% in host type B.

### Narrative description of comparative studies

We identified three comparative studies. The study from Romano et al. [13] compared the results of three different dead space management strategies. Patient groups were matched based on patient age, host grade and disease stage and followed up for between 12 and 36 months (mean 21.8). The recurrence rates for bioactive glass versus antibiotic-loaded calcium hydroxyapatite versus antibiotic-loaded demineralised bone matrix were similar (no recurrence in 87.5 to 89.8%, *N* = 20 to 25 per group; MCMS = 71).

The study by Chang et al. [10] compared the use of debridement alone (*N* = 40) with debridement and antibiotic-loaded calcium sulphate pellets (*N* = 25). Forty percent of patients experienced recurrence with debridement alone compared to 20% with dead space management. This study had, however, one of the lowest MCMS (quality) scores (62).

The third comparative study from Simpson et al. [4] reviewed the impact of extensive versus marginal debridement with dead space management compared to minimal debridement and no dead space management strategy. Wider debridement had a higher non-recurrence rate (100%, *N* = 8) compared to marginal debridement (71.4%, *N* = 21) [both with dead space management] versus minimal debridement (0%, *N* = 4: all host type C) [MCMS = 75].

### Narrative description of largest studies

Only two studies included more than 50 patients for any particular treatment approach used. These studies were of prospective design, from the same centre and had the highest quality scores (MCMS = 79 and 83) [14, 15]. The study by Ferguson et al. reported on 128 patients with chronic long bone osteomyelitis treated with debridement and dead space management using Osteoset-T pellets (calcium sulphate plus tobramycin). There was no evidence of recurrence during follow-up in 89.8%. The study of McNally et al. used Cerament-G pellets (calcium-sulphate hydroxyapatite plus gentamicin) for dead space management following debridement in 78 patients with chronic long bone osteomyelitis. All of these patients had stage 3 or 4 disease, unlike the previously discussed study where 10% of their subjects had only stage 1–2 disease. This second study found no evidence of recurrent infection in 97.4% of subjects during follow-up, but had shorter lower-end (12 versus 16 months) and mean follow-up (19.5 versus 44 months) periods.

## Discussion

Although the individual size of studies and overall number of patients were small, and most studies of uncontrolled, non-comparative design, some clinically useful conclusions can be made. Debridement alone without adequate dead space management is a sub-optimal approach in the management of chronic osteomyelitis. There is no clear evidence that any one approach to dead space management is, however, superior (i.e. biologic versus non-biologic) and different approaches within each of these groups were comparable. We were also able to identify a number of deficiencies that, if rectified, could improve the reporting of similar studies in the future.

Similar success rates have been reported for multi-stage procedures (70 to 95%) [16], but the single-stage approach to chronic osteomyelitis has potential advantages for both patients and healthcare systems, for example, a reduction in post-operative morbidity with less time off work, and financial savings through shorter hospital stay, potentially less antibiotic use and a reduction in theatre time through multiple surgeries.

When comparing the results for biological dead space management techniques in single-stage procedures with those previously reported for multi-stage procedures, the results are equivalent. One large study reviewing the use of microvascular free flaps in the management of dead space in a multi-stage procedure reported a non-recurrence rate of 95.8% [17]. Other studies where muscle flaps were used as void fillers in multi-stage procedures found rates of between 81 and 100% for non-recurrence of infection within the specified follow-up period [18, 19]. Our results in single-stage procedures where muscle flaps were used for dead space management identified a non-recurrence rate of 95.1%.

On review of the use of antibiotic-loaded polymethyl methacrylate (PMMA) beads, in the management of dead space, the results were slightly superior in the multiple-stage surgery literature. One study described debridement and insertion of vancomycin-impregnated PMMA beads, followed by a second procedure 6 weeks later to remove the beads and perform further debridement as required. Their non-recurrence rate was calculated to be 88.5% [20]. Another reported a similar protocol where the second stage of bead removal was carried out 3 weeks following the initial debridement, with a non-recurrence rate of 87.8% [21]. The single-stage surgeries identified in this study achieved a non-recurrence rate of 79.3% when PMMA beads were selected as the void filler. Due to the non-degradable nature of PMMA beads, they will always have to be removed from the body, which in the single-stage surgery cohort was achieved by leaving the row of beads proud of the skin to allow for gradual removal without a second surgical procedure [4]. It is possible that this opening in the soft tissues to allow for bead removal resulted in a higher recurrence rate in the patients in our study compared to two-stage procedures where PMMA beads were used.

With regard to other non-biologic methods of dead space management, there are no published reports of multi-stage surgeries for comparison. The bio-degradable nature of newer antibiotic-impregnated void fillers makes them highly suitable for use in single-stage procedures. Antibiotics can be delivered locally at high concentrations with no need for removal by a second surgical procedure.

Many studies included mixed patient groups with data on infected non-union cases presented with chronic osteomyelitis cases. These disease processes and their treatment strategies are very different, and the skeletal stability required when treating non-union makes a single-stage procedure more challenging. We extracted data for the “pure” chronic osteomyelitis cases as accurately as possible from these mixed data sets to obtain information specific to this condition. The inclusion of cases of infected non-union in many of these studies may well have skewed overall study results. In the future, conclusions that are more meaningful will be achieved by studying and presenting data on these disease entities separately.

The reporting of the microbiology samples obtained during the procedures was of variable quality throughout the literature. Three of the 13 articles included did not report any microbiological results. Of the 10 papers that did present microbiological findings, 19% of patients did not have a positive culture throughout their entire course of treatment. This emphasises the potential importance of non-culture-based microbiological tests, such as PCR, in optimally targeting antibiotic therapy to infecting pathogens. The diagnosis of chronic osteomyelitis in culture-negative cases was therefore based on clinical presentation, imaging, operative findings and histology. The most common organism isolated was *Staphylococcus aureus* (35.2%), but polymicrobial, Gram-negative and *Pseudomonas* spp. infections were also frequent. This emphasises that the local and systemic delivery of antibiotics that cover only Gram-positive bacteria may be inadequate for some patients.

There was minimal data reported regarding systemic antibiotics prescribed post-operatively, although many articles stated that regimens were tailored to an individual’s culture results. The duration of antibiotic therapy following the single-stage procedures was also absent from many reports. Some studies reported using the traditional regimen of 2 weeks of intravenous followed by 4 weeks of oral antibiotic therapy (6 weeks total). Another study reported the use of antibiotic therapy for only 48 h following the procedure, and another described oral antibiotic treatment for up to 4 months post-operatively. This large variation in practice makes it impossible to draw conclusions on the effectiveness of post-procedure antibiotic use but highlights the importance that future studies report this in detail.

The tibia was the most reported site of infection with some studies specifically reporting tibial disease. Due to the relative poor blood supply to the tibia compared with other long bones, we would have expected the highest recurrence rate at this anatomical location, but reported recurrence was low and less than that at other sites.

A large proportion of patients were healthy type A hosts (44%), which may have accounted for the high reported success rates. The high reported recurrence rate in host type C patients may have been exaggerated by the type and complexity of procedures undertaken in different host types. For example, because of concerns about surviving a more extensive procedure, one study reported only performing minimal debridement in host C patients with all patients then suffering a recurrence of infection.

## Limitations

Some limitations of these reviewed studies are discussed above. Additionally, follow-up periods varied vastly between studies (from 12 months to 27 years) making comparisons of outcomes challenging, particularly as osteomyelitis can recur many years after surgery. There was little data from low- and middle-income countries where the burden of disease is likely to be greatest, but resources available for treatment the lowest. Some of the studies, including those of the highest quality, were from national centres of excellence and the external validity of such studies is therefore debatable. Future studies should avoid mixing different patient cohorts and report in detail the nature of the host, how the diagnosis was made, microbiology results (including the presence of resistance), what antibiotic therapy was administered (including the route and duration) and outcomes at defined follow-up time points (e.g. 1 year) for all patients.

## Conclusions

We have identified that a wide-range of apparently successful single-stage procedures are being performed for chronic osteomyelitis, mostly in high-income countries. Debridement alone leads to disease recurrence significantly more frequently than approaches using dead space management. The results of biologic and non-biologic methods were comparable. Further research should ideally be multi-centred with larger numbers of patients for each approach being compared. A national infection register with a basic minimum dataset to extract meaningful outcomes may be helpful.

## Supplementary information

**Additional file 1.** PRISMA checklist.

## Data Availability

Available upon request

## Abbreviations

COM: Chronic osteomyelitis
MCMS: Modified Coleman Methodology Score
MRSA: Methicillin-resistant *Staphylococcus aureus*
PMMA: Polymethyl methacrylate
PRISMA: Preferred Reporting Items for Systematic Reviews and Meta-Analyses
